# Age-related differences in learning, memory and brain plasticity in workers of the common wasp, *Vespula vulgaris*

**DOI:** 10.1242/jeb.251673

**Published:** 2026-01-05

**Authors:** Anneline Mattens, Hannelore Christiaens, Kamiel Debeuckelaere, Viviana Di Pietro, Helena Mendes Ferreira, Fabio Santos do Nascimento, Cintia Akemi Oi, Tom Wenseleers

**Affiliations:** ^1^ Laboratory of Socioecology and Social Evolution, KU Leuven, 3000 Leuven, Belgium; ^2^ Laboratório de Comportamento e Ecologia de Insetos Sociais, Universidade de São Paulo, Ribeirão Preto - SP, CEP 14040-900, Brazil; ^3^ Centre for Biodiversity and Environment Research, University College London, London WC1E 6BT, UK

**Keywords:** Associative learning, Cognition, Neuroanatomy, Ageing, Social insects, Eusocial wasps

## Abstract

Learning, memory and brain plasticity are thought to play an important role in regulating behavioural roles in social insects, as workers perform different tasks as nurses, builders, foragers and defenders. However, it remains challenging to disentangle whether neural changes regulate behaviour or arise as a consequence of it. While cognition has been extensively studied, especially in honeybees, the variation of cognitive traits remains poorly understood in social wasps. Here, we investigated age-related changes in learning, memory and neuroanatomy in workers of the common wasp, *Vespula vulgaris.* We developed a Y-maze to test differential conditioning and memory of wasps and later visualised the brains using a high-resolution micro-computed tomography imaging. We found that younger individuals exhibited slower decision making yet made more accurate decisions compared with older individuals, revealing a pronounced speed–accuracy trade-off. Short-term memory showed only a slight decline with age. Neuroanatomical image analysis revealed that, despite a reduction in overall brain volume, key major neuropils involved in sensory processing and learning, such as mushroom bodies, optic lobes and antennal lobes, increased in relative volume with age. These findings corroborate with studies in bees and provide novel insights into how ageing influences cognitive function and brain structure in wasps.

## Teaser

**Highlighted Article:** This first integrated analysis of age, cognition and brain plasticity in *Vespula vulgaris* suggests an age-related speed–accuracy trade-off in learning and an increase in relative volume of major neuropils with age.

## INTRODUCTION

Social insects forage in highly dynamic and unpredictable environments, where food availability fluctuates rapidly as a result of displacement or depletion (Manattini et al., 2024). To cope with these challenges, they have evolved remarkable cognitive abilities, with learning and memory at the core of their behavioural flexibility (Agrawal, 2001; Giurfa, 2015; Richter, 2000; Tibbetts, 2002; Tibbetts et al., 2018). Associative learning allows individuals to link stimuli with rewards or punishments (El-Sayed et al., 2018) while spatial learning supports navigation using visual and olfactory landmarks (Moreyra et al., 2017). Together, these abilities enable social insects such as wasps (Gong et al., 2019; Richter, 2000), ants (Fenli et al., 2025; Hölldobler and Wilson, 1990) and bees (Giurfa, 2007, 2015; Kheradmand et al., 2020; Leadbeater and Chittka, 2007; Menzel and Giurfa, 2001) to efficiently exploit resources and adapt to changes in their environment, and are considered key components of their ecological and evolutionary success.

Variation in cognitive performance is shaped not only by ecological factors but also by ageing (Tonoki and Davis, 2015). In solitary insects such as the fly *Drosophila melanogaster*, senescence is typically associated with declines in learning and memory driven by synaptic degeneration, oxidative stress and altered neurotransmission (Iliadi and Boulianne, 2010; Tonoki and Davis, 2015). By contrast, eusocial insects often display extended lifespans and division of labour, such that cognitive performance is shaped as much by behavioural role as by chronological age (Gage et al., 2020; Giraldo et al., 2016; Scheiner and Amdam, 2009). A major driver of this role-related variation is temporal polyethism, the age-related division of labour that structures colony life (Siegel et al., 2013). Workers typically begin adult life performing tasks within the nest before transitioning to foraging, a shift often accompanied by changes in learning, memory and sensory responsiveness (Gage et al., 2020; Scheiner and Amdam, 2009; Siegel et al., 2013). For example, in honeybees, foragers exhibit heightened sensitivity to visual and olfactory cues compared with nursing workers (Tsuruda and Page, 2009), with performance further modulated by both intrinsic factors (e.g. hormonal state) and extrinsic factors (e.g. environmental conditions) (Brothers, 2021; Fahrbach and Robinson, 1996). Taken together, these findings suggest that the evolution of social complexity may buffer some age-related declines while amplifying role-specific cognitive specialisations, raising the question of whether cognitive ageing represents a conserved constraint or an adaptive trajectory in social insects.

Such behavioural changes observed in social insects are closely tied to learning and memory processes. Short-term memory (STM) forms rapidly after conditioning and typically decays within hours, whereas long-term memory (LTM) requires repeated, spaced training and can persist for days or longer (DeZazzo and Tully, 1995; Giurfa, 2015; Moreyra et al., 2017). In honeybees, associative memory underpins odour and colour discrimination as well as complex long-term learning (Chittka, 1998; Dyer and Garcia, 2014; Giurfa, 2004; Menzel, 1999), but performance varies with age and task: long-lived winter bees largely retain memory, whereas foragers show subtle impairments or division-of-labour effects on olfactory learning (Behrends and Scheiner, 2010; Maleszka et al., 2009). Ants also rely on robust visual and olfactory memories to navigate and exploit resources, supported by well-developed mushroom bodies (Gronenberg et al., 1996; Hölldobler and Wilson, 1990). Comparable flexibility occurs in wasps with solitary and eusocial lifestyles. For instance, the parasitoid jewel wasp *Nasonia vitripennis* retains olfactory associations for several days, which facilitates the localisation of new hosts (Schurmann et al., 2012), the social paper wasp *Mischocyttarus* show both colour reward learning and sex- and age-dependent differences (da Silva et al., 2023), and the paper wasp *Polistes fuscatus* can remarkably recognise conspecific faces (Sheehan and Tibbetts, 2008; Tibbetts et al., 2021). In fact, neurobiological studies are beginning to reveal the mechanisms underlying visual individual recognition in paper wasps, while genomic analyses point to recent selection shaping their cognitive abilities (Tumulty et al., 2023). In the eusocial species *Apis mellifera* and *Vespula vulgaris* it was shown that the individuals are even able to discriminate human faces (Avarguès-Weber et al., 2018). Collectively, these studies highlight that advanced cognitive abilities are widespread across Hymenoptera and that they are shaped by ecological as well as social pressures.

The neural basis of cognitive abilities in insects lies in a highly structured brain, where specialised neuropils process and integrate sensory information (DeZazzo and Tully, 1995; Gronenberg et al., 2007). The antennal lobes (AL; olfaction), optic lobes (OL, including medulla and lobula; vision), central complex (CX; locomotor control) and mushroom bodies (MB; sensory integration, associative learning and memory) are particularly important, with the last of these supporting associative learning and memory (Ehmer and Gronenberg, 2002; Habenstein et al., 2023; Hansson and Anton, 2000; Heisenberg, 2003; Rother et al., 2021; Rybak et al., 2010). Social insects typically invest heavily in the MBs, reflecting their reliance on spatial cognition and chemical communication (Fahrbach, 2006; Heisenberg, 2003). In honeybees and bumblebees, MBs expand as workers age and transition to foraging, reflecting experience-dependent synaptic reorganisation rather than neurogenesis (Fahrbach et al., 1998; Fahrbach and Robinson, 1996; Farris et al., 2001). Comparable plasticity has been observed in the paper wasps *Polistes* and *Mischocyttarus*, where social experience and dominance behaviour influence MB volume and regions associated with visual processing (Jernigan et al., 2021; Molina and O'Donnell, 2008; O'Donnell et al., 2007). The same pattern was observed in solitary alkali bees (*Nomia melanderi*), with experience and not age driving MB expansion, reinforcing that adult experience is a conserved determinant of neural investment across Hymenoptera (Hagadorn et al., 2021). From a neuro-ecological perspective, variation in cognitive demands across behavioural roles is expected to drive differential brain investment, as brain tissue is metabolically costly and colony-level selection may favour optimised allocation tailored to caste- or task-specific functions (O'Donnell et al., 2018). However, neural plasticity could also arise through regulatory changes and intrinsic developmental or genetic programmes that guide brain maturation, as experience-independent phases of adult MB growth have also been documented in honeybees (Fahrbach et al., 1998). Because these reflective and regulatory changes often overlap, disentangling whether age- and role-related brain differences regulate behaviour or arise as its consequence remains a central challenge.

Compared with honeybees, where age- and role-related changes in cognition are well studied, far less is known about such processes in highly eusocial wasps such as *V. vulgaris*. Previous studies showed that wasps are capable of learning and memorising associations: *V. vulgaris* and *V. germanica* foragers perform specialised orientation flights to gather spatial, visual and olfactory information about food sources (Collett and Rees, 1997; D'adamo and Lozada, 2003; Lozada and D'Adamo, 2014), often forming long-lasting associations after just a single visit (D'adamo and Lozada, 2007; Moreyra et al., 2017). When food is displaced, they first search the original site before adjusting to new environmental cues (Lozada and D'Adamo, 2011, 2014; Manattini et al., 2024; Wilson-Rankin, 2015), highlighting behavioural plasticity. Wasps also exhibit robust associative learning, readily linking colours and odours with rewards (D'adamo and Lozada, 2009; El-Sayed et al., 2018; Yossen et al., 2020). Furthermore, Y-maze experiments with *V. germanica* and hornets (*Vespa velutina nigrithorax* and *Vespa crabro*) showed that they not only are capable of acquiring colour–reward associations but also can reverse them when contingencies shift (Lacombrade et al., 2023; Moreyra and Lozada, 2021), a flexibility comparable to the visual learning capacities documented in nectar-feeding bees (Dyer and Howard, 2023).

Here, we fill the gap in the existing knowledge on learning and memory in wasps, by testing age-related differences in learning and STM in *V. vulgaris* workers, and by examining their neuroanatomy using high-resolution micro-computed tomography (micro-CT). Based on findings from bees and other wasps, we hypothesised that newly emerged workers would exhibit lower cognitive performance, whereas older foragers would show enhanced learning and memory associated with structural changes in key brain regions, particularly the MBs.

## MATERIALS AND METHODS

**Fig. 1.**
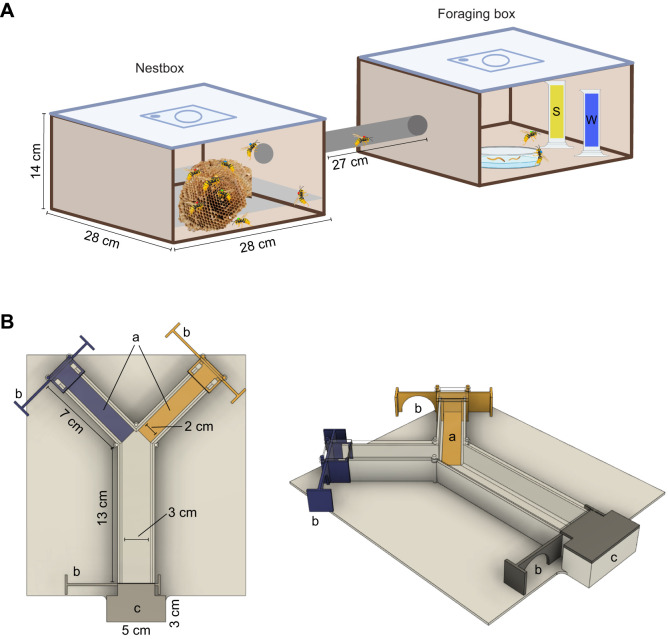
**(A) Laboratory colony setup for *Vespula vulgaris*.** Each colony was housed in a wooden nestbox containing a single comb, the queen, 50–100 older workers and marked newly emerged workers. The nestbox was connected via a plastic tube to a foraging box supplied *ad libitum* with sugar water (S), water (W), mealworms and nesting material. Created in BioRender by Christiaens, H., 2025. https://BioRender.com/l78wfvo. This figure was sublicensed under CC-BY 4.0 terms. (B) The 3D printed Y-maze including interchangeable coloured sides (a), sliding doors (b) and a holding chamber (c). The top is covered with Plexiglas, allowing observation of behaviour and easy removal for cleaning in between runs.

Seven *Vespula vulgaris* (Linnaeus 1758) nests were collected in the region of Leuven (Belgium; 50°52′46″N, 4°42′3″E) during the summer of 2024. Colonies were kept in wooden nestboxes (Fig. 1A) under standard conditions (28°C and 12 h:12 h light:dark cycle) in the laboratory. Each nestbox was covered by a dark cloth and connected to a foraging box where water, sugar water (50:50), mealworms and nesting material were given *ad libitum*. Each nestbox contained a single comb with emptied cells to allow egg laying, while larvae of different developmental stages and pupae were kept, allowing emergence, to retain natural transitions. The queen and 50–100 older workers were introduced into the nest box to promote natural behaviour. Over the following 3 days, newly emerged individuals from the remaining combs were checked and collected daily (newly emerged per colony: *n*=160–190, mean *n*=175), marked with a paint marker (Uniball Paint Marker PX-21) and returned to their respective colonies. These individuals were subsequently used for both associative learning trials and neuroanatomical comparisons.

### Learning and memory trials

Associative learning and STM were assessed in 176 workers of known ages: newly emerged (*n*=47), 3 days old (*n*=44), 6 days old (*n*=34), 9 days old (*n*=30) and 12 days old (*n*=21). Under our laboratory conditions, adult *V. vulgaris* had a short life span, surviving only around 12–14 days, so 12-day-old wasps were selected as the oldest group to capture the late phase of adult life in our setup. Age categories (0, 3, 6, 9 and 12 days) were chosen to represent the in-nest (0–3 days) phase versus onset and progression of foraging (6–12 days) (Ferreira et al., 2023; Hurd et al., 2007).

Differential conditioning was performed using a custom 3D printed Y-maze with interchangeable coloured arms (blue and yellow), colours used in previous wasp learning experiments (Howard and Dyer, 2024) (Fig. 1B). Prior to conditioning, age-specific wasps were removed from the nestboxes and placed in isolation where they underwent starvation for 2 h to increase motivation. They were then transferred to the Y-maze's holding chamber to acclimatise for 5 min before starting the trials. Each series of trials began with an exploration run, in which wasps were allowed to walk freely in the maze, without any food reward present. Their first choice of side (left or right) and colour (yellow or blue) was recorded to assess potential side or colour biases.

Following this, each wasp underwent six consecutive conditioning trials (see fig. S1, example movie 1 in https://doi.org/10.17632/wxxkwjk9hm.4). In each trial, sugar solution (50:50) was placed in an Eppendorf cap at the end of one coloured arm, serving as the positively conditioned stimulus (CS+). The other arm contained water, acting as a neutral conditioned stimulus (CS). Each age group included at least 10 individuals per colour condition (see table S1 in https://doi.org/10.17632/wxxkwjk9hm.4). The colour-reward pairing (blue or yellow as CS+) remained constant throughout the six trials for each individual. To control for spatial learning, the positions left (L) and right (R) of the coloured arms were pseudo-randomised across four switching sequences (Y1/B1: LRRLRL, Y2/B2:RRLLLR, Y3/B3:LRLLRR or Y4/B4:RLRLRL), assigned per treatment group (see table S2 in https://doi.org/10.17632/wxxkwjk9hm.4).

The time taken to find the reward was measured from the moment the wasp entered the maze. If the wasp failed to locate the reward within 5 min, the trial was ended. Wasps that failed to complete two consecutive trials were removed and replaced with a new individual of the same age group (see table S4 in https://doi.org/10.17632/wxxkwjk9hm.4). Upon finding the reward, the wasp was allowed to feed for 3 s before the reward was removed. It was then given the chance to explore the maze to find the non-rewarding feeding cup containing water. Once the wasps returned to the starting chamber, the sliding door was closed and a 5 min rest period began. This procedure was repeated for the remaining five conditioning trials. After the final conditioning trial, a 2 h break was initiated, preceding two STM trials. These STM trials followed the same maze protocol as the conditioning phase, except that no reward was provided. Empty feeding cups were placed in the maze to check for antennation, which was used as an indicator of active search behaviour. Colour and side choice, as well as the time taken to reach the feeding cup at the trained reward side was again recorded.

### Age-related neuroanatomy

**Fig. 2.**
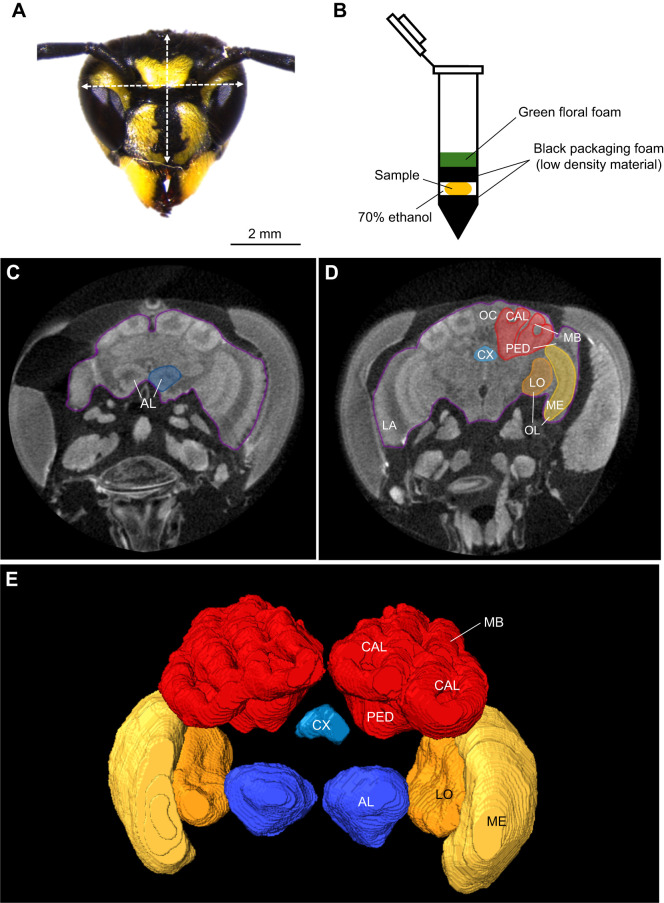
**Overview of micro-CT preparation and analysis of *V. vulgaris* worker brains.** (A) Frontal view of a *V. vulgaris* worker head showing landmarks for head width and height measurements, taken above the antennal sockets and from vertex to clypeus margin, respectively. (B) Micro-CT sample preparation in an Eppendorf tube, with heads submerged in 70% ethanol and stabilised using low-density black soundproofing material and green floral foam to prevent movement without interfering with image quality. (C) Frontal 2D slice at the level of the antennal lobes (AL). (D) Frontal 2D slice showing the position of the central brain within the head capsule, with neuronal tissues labelled, including the lamina (LA) and three ocellar synaptic plexi (OC), of which only one is visible. The purple contour outlines the total brain area used for total brain volume calculations. (E) 3D surface reconstruction of the brain from a frontal perspective, with neuronal tissue between the central complex (CX, light blue) and AL (dark blue) removed for visibility. Other neuropils are: optic lobe (OL) including the lobula (LO, orange) and medulla (ME, yellow), mushroom body (MB, red) including two calyces (CAL) and a peduncle (PED).

Additionally, we investigated age-related brain plasticity. Six wasps per age category (30 individuals in total) were randomly selected following the completion of the memory trials. The wasps were decapitated, their mandibles removed, and a small opening was made in the posterior region of the head capsule to improve reagent infiltration during subsequent preparation steps. All heads were photographed in frontal view using a Leica stereomicroscope connected to a digital camera (Leica MSV266) using the Leica Application Suite program. To estimate average head capsule depth (mm), we additionally photographed 31 randomly chosen heads in lateral view (see table S5 in https://doi.org/10.17632/wxxkwjk9hm.4). Images were analysed using Fiji (ImageJ 1.54p) (Schindelin et al., 2012). Head width (mm) was measured at the widest point above the antennal sockets and head height (mm) was measured from the centre of the vertex to the bottom tip of the anchor shape on the clypeus, at the level of the lower eye margin (Fig. 2A). The measurements were then used to calculate the head capsule volume (mm^3^) using the formula for an ellipsoid (O'Donnell, 2019):
(1)

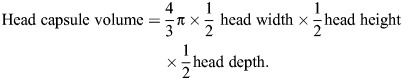
Heads were then fixed in 4% paraformaldehyde (PFA) until further processing for micro-CT imaging. Seven days prior to scanning, heads were washed twice in 1× PBS for 30 min each, then submerged in a 5% phosphotungstic acid (PTA) solution containing 3% DMSO to enhance contrast of brain tissue. Heads were scanned in 70% ethanol within Eppendorf tubes, stabilised using low-density black soundproofing material and green floral foam (Fig. 2B). Imaging was performed using a Skyscan 1272 X-ray micro-computed tomography microscope at the FIBEr facility (KU Leuven, Belgium). Scanning settings included a 1 mm aluminium filter, 2016×1344 pixel resolution, 6 µm voxel size, 0.3 deg rotation steps, 3-frame averaging, 89 kV voltage and 112 µA current.

Micro-CT scans of the heads were processed and analysed using Amira software (v.5.3.3). Regions of interest of the brain, including OLs (medulla and lobula), antennal lobes, MBs, central complex and total brain area, were manually traced for annotation. A 3D reconstruction of the brain was created, and volume measurements (mm^3^) were obtained for each region (Fig. 2C–E).

### Data analysis

All statistical analyses were performed in R (v.4.4.2) (http://www.R-project.org/) and graphs were made using *ggplot2* (Wickham, 2016).

To assess whether wasps exhibited pre-existing biases toward colour (blue or yellow) or spatial orientation (left or right) prior to conditioning, we fitted two binomial generalised linear models (GLMs), using the *lme4* package (Bates et al., 2015). The response variable was the first colour choice or side choice, with age (scaled) as a continuous predictor to capture potential developmental effects on learning. For interpretability, predicted probabilities were computed at representative ages (0, 3, 6, 9 and 12 days) by inverse-logit transformation of the fitted log-odds. These model-derived probabilities incorporate the estimated age effect and offer a smoothed representation of the expected choice behaviour. Model fit was assessed using residual diagnostics, and Tukey *post hoc* comparisons between age groups and within age group comparison with random choice (0.5) were conducted using the *emmeans* package (https://cran.r-project.org/web/packages/emmeans/index.html).

To assess learning performance across different age groups, binomial generalised linear mixed models (GLMMs) were fitted using the *glmer* function in *lme4* package (Bates et al., 2015), with choice correctness (correct/incorrect) as the response variable. Fixed effects included the scaled continuous variables age, trial and time, as well as the categorical variable trained colour and the interaction between time and age. An offset term was added to control for any intrinsic colour bias. Colony and individual ID were included as random effects.

To analyse changes in search time across learning trials while accounting for censored observations (i.e. trials in which the reward was not located within 300 s), we carried out a survival analysis. Both accelerated failure time models (*survival* package; Therneau and Grambsch, 2000) and flexible parametric survival models (*flexsurv* package; Jackson, 2016) were tested, with the generalised gamma model providing the best fit.

STM performance was analysed using a similar binomial GLMM approach to that for learning performance, with choice correctness (binary: correct/incorrect) during memory trials as the response variable. To ensure that learning occurred, only individuals with 4 or more correct choices during the conditioning phase were included (see table S3 in https://doi.org/10.17632/wxxkwjk9hm.4). Alternative filters (≥5 or 6 correct choices) were tested but yielded qualitatively similar results. Fixed effects included age, trial, time and colour. We also tested whether the predicted probability of correct choice for each age group significantly differed from random choice (0.5) using the *emmeans* package (https://cran.r-project.org/web/packages/emmeans/index.html).

Finally, to assess age-related changes in brain structure, we analysed absolute and relative neuropil volumes using linear models (lm), with age as a continuous predictor of the performance variable. For models of absolute volume, head capsule volume and total brain volume were included as covariates to control for body size. For models of relative volume, age and head capsule volume were used as predictors, as the response variable was already normalised by total brain volume. Colony identity (three levels) was included as a random factor in the candidate GLMMs and evaluated by AICc. Models retaining the colony term had higher AICc and a near-zero variance component, and fixed-effect estimates and inferences were unchanged. Accordingly, the AICc-supported GLMMs without the colony term are reported as the final models. Model assumptions were checked via residual diagnostics. Where assumptions were violated, we applied log-transformations (e.g. for absolute lobula volume) or spline fits to account for non-linearity (e.g. for absolute volumes of the central complex and total brain, and relative volume of the medulla).

### AI tools

Artificial intelligence tools (ChatGPT, OpenAI) were used to assist with R code development and troubleshooting as well as for grammar checking of the manuscript text. All scientific content, analyses and interpretations were designed, conducted and verified by the authors. The authors subsequently reviewed and edited the content as necessary and take full responsibility for the publication’s final content.

## RESULTS

### Colour and side bias

**Fig. 3.**
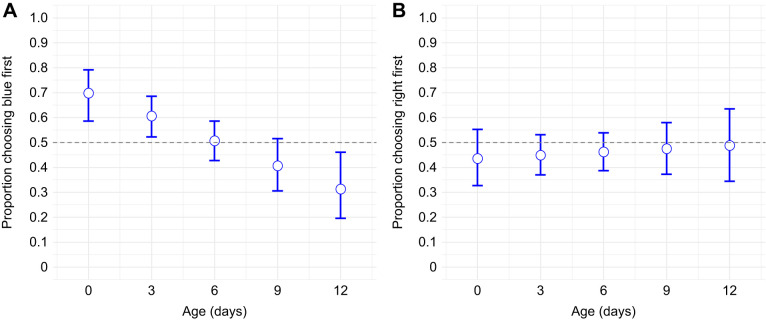
**Inherent bias in colour and side preference across ages in *V. vulgaris* workers.** (A) Younger wasps (0 and 3 days, *n*=47 and *n*=44, respectively) showed a significant bias toward blue (binomial GLM, *P*<0.001), while 12-day-old wasps (*n*=21) preferred yellow (bias against blue, *P*=0.014). In contrast, 6-day-old and 9-day-old wasps (*n*=34 and *n*=30, respectively) showed no significant colour preference (binomial GLM, *P*=0.091). (B) No inherent bias was observed in side choice across any age group (*P*=0.64). Error bars indicate 95% confidence intervals. Significance levels based on comparisons with random choice (0.5).

Each wasp was first allowed to perform an exploratory run to test for inherent bias toward either colour or side in the Y-maze. Age had a significant effect on initial colour choice (GLM: *z*=−3.412, *P*<0.001). Out of the 175 wasps tested, 95 chose blue (54%) and 80 chose yellow (46%). To test whether colour preference changed with age, we analysed the proportion of wasps choosing blue across our five age groups (0, 3, 6, 9 and 12 days; Fig. 3A). Initial colour preference significantly deviated from chance (0.5) in an age-dependent manner. At day 0, 69.8% of wasps chose blue as their first choice (*P*<0.001). Similarly, 3-day-old wasps also showed a significant bias toward blue (60.7%, *P*=0.014). In contrast, 6- and 9-day-old wasps showed no significant colour preference (6 days: 50.7%, *P*=0.87; 9 days: 40.6%, *P*=0.091). Interestingly, 12-day-old wasps showed a significant preference for yellow, with only 31.3% of wasps choosing blue as their first choice (*P*=0.014). In contrast, for side choice (left of right arm of the Y-maze) (*z*=0.47, *P*=0.64), no age group differed from chance (all *P*>0.2; Fig. 3B). Because we observed a significant age-specific bias in colour preference, we corrected for this in the models for learning and memory.

### Associative learning

**Fig. 4.**
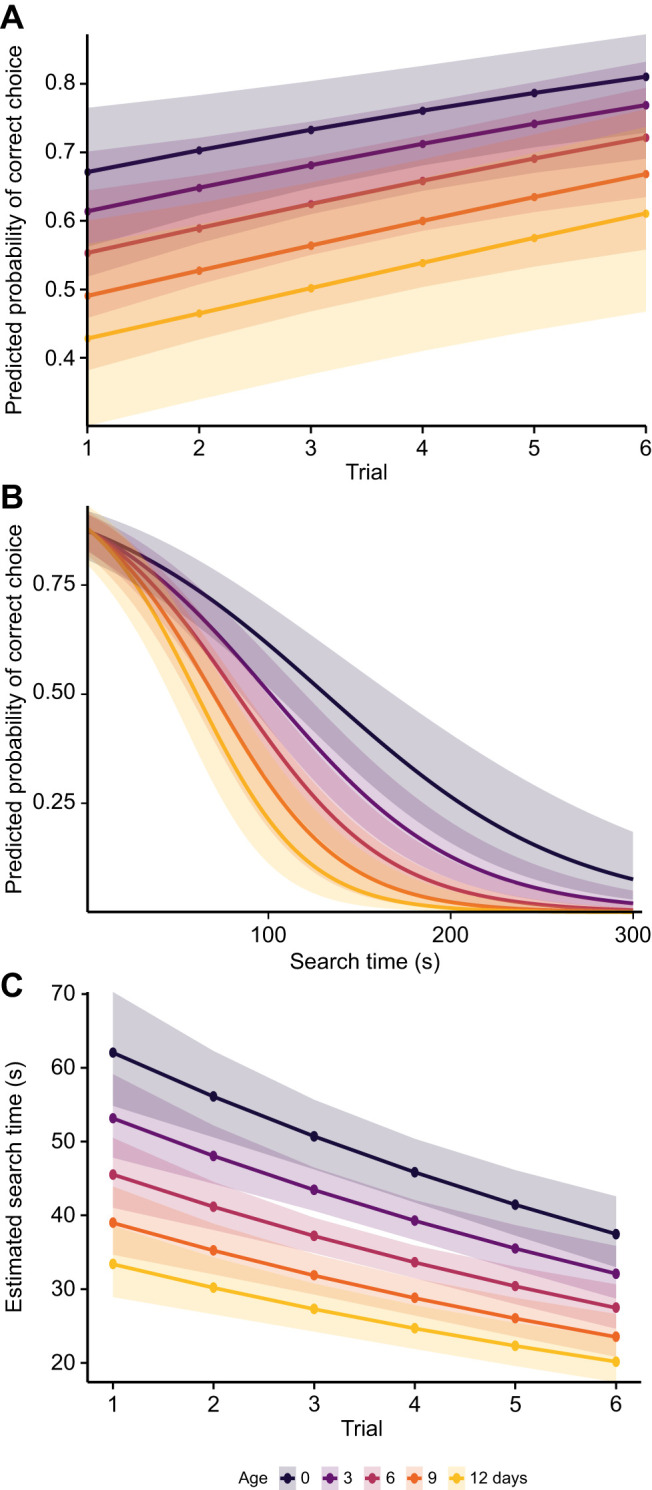
**Learning performance and search time during Y-maze conditioning trials in *V. vulgaris* workers (*n*=176).** Shaded areas indicate 95% confidence intervals. (A) Predicted probabilities of correct colour choice from a binomial GLMM show that accuracy increased with trial number but was lower in older workers (*P*<0.01). (B) Probability of correct choice declined with increasing search time (*P*<0.001), with a stronger negative effect in older individuals (*P*<0.01). (C) Estimated search times from a parametric survival analysis decreased significantly across trials (*P*<0.001) and with age (*P*<0.001). Search time was measured from departure from the holding chamber to reward consumption.

Learning performance was evaluated across six conditioning trials using a binomial GLMM. The probability of making a correct choice (i.e. selecting the rewarded colour) increased significantly over trials (β±s.e.=0.25±0.08, *P*<0.01), indicating that the wasps learned the colour–reward association over time. However, overall performance declined with increasing age (β=−0.34±0.12, *P*<0.01; Fig. 4A). Trained colour (yellow or blue) did not have a significant influence on the probability of a correct choice (*P*=0.18).

Longer search durations were associated with a significantly lower probability of making a correct choice (β=−1.61±0.16, *P*<0.001), indicating that increased decision time may reflect or contribute to impaired task performance. This relationship was moderated by age as the negative effect of search time on accuracy was significantly stronger in older wasps (β=−0.45±0.14, *P*<0.01; Fig. 4B). While younger wasps exhibited a higher overall probability of correct responses, older individuals demonstrated a more pronounced decline in choice accuracy during longer searches. Random effects in the model confirmed substantial individual- and colony-level variation, with individual ID accounting for a large proportion of the variance.

To further investigate the predictors of search time, we fitted a parametric survival model using a generalised gamma distribution (trials 1–6). Search time decreased significantly with age (β=−0.05±0.0072, *P*<0.001), indicating that older wasps completed the task more quickly (Fig. 4C). Similarly, search time decreased significantly across repeated trials (β=−0.10±0.018, *P*<0.001), suggesting faster performance of all ages with repeated exposure. Trained colour had no significant effect (*P*=0.72). Interestingly, wasps that made a correct choice had significantly longer search times than those that made incorrect choices (β=0.60±0.03, *P*<0.001), suggesting a speed–accuracy trade-off during decision making.

### STM

To assess memory performance following conditioning, we analysed trials 7 and 8 [memory trials (MT) 1–2], conducted without reward, using a GLMM with a binomial error structure and individual ID as a random intercept. Only wasps that had achieved at least four correct choices during the conditioning phase were included (*n*=216 observations from 108 individuals). Out of these 108 wasps, 62 (57.4%) had good memory (two correct choices), 37 (34.3%) had neutral memory (one correct choice) and 9 (8.3%) had bad memory (two incorrect choices).

**Fig. 5.**
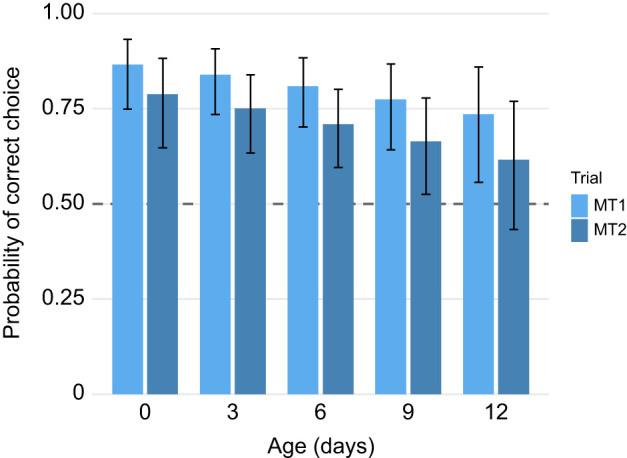
**Memory performance across age groups in *V. vulgaris*.** Only individuals with ≥4 correct choices during conditioning were included (*n*=108). Probability of correct choice was overall higher than chance (binomial GLMM, 76.6%, *P*<0.0001), indicated by the dashed line (50% chance level). No significant differences in memory performance were detected across age groups (*P*=0.111) or memory trial (MT; *P*=0.125). All ages were significantly different from random choice (0, 3, 6 days old: *P*<0.001; 9 days old: *P*=0.04), except 12 day old (*P*=0.24). Error bars represent 95% confidence intervals.

To determine whether memory performance exceeded random choice levels, we compared the observed number of correct choices during memory trials (MT 1–2) with chance level (50%) using a binomial test. Wasps performed significantly better than chance in memory trials, with 160 correct choices out of 216 (*P*<0.0001). A model-based estimate using a GLMM confirmed this result, with an overall predicted probability of correct choice of 76.6%. The probability of making a correct choice during these memory trials was not significantly affected by age (β=−0.29±0.18, *P*=0.111), trained colour (β=0.16±0.18, *P*=0.366) or trial number (β=−0.28±0.18, *P*=0.125) (Fig. 5). All ages were significantly different from random choice (0, 3, 6 days: *P*<0.001; 9 days: *P*=0.04), except 12-day-old wasps (*P*=0.24).

Search time was a strong predictor of performance with longer search durations being associated with a significantly reduced probability of making a correct choice (β=−1.20±0.27, *P*<0.001). Random effects accounted for individual-level variation (σ^2^=0.022). These results suggest that rapid decisions during memory tests were more likely to be correct, while longer latencies to choose reflected uncertainty or memory decay. As these results are based on the group of wasps making four or more correct choices during the conditioning trials, we also checked for a stricter filter only including wasps making five or six correct choices. This resulted in a total of 61 (4 bad, 18 neutral and 39 good) and 25 wasps (4 neutral and 21 good), respectively. However, this only decreased the number of wasps performing badly during the memory trials but did not affect the outcome.

### Neuroanatomy

All micro-CT images of brains were reconstructed into 3D models comprising six distinct neuropils, which were used to perform volume measurements. We report both absolute and relative brain region volumes. Absolute volumes provide baseline anatomical information and capture individual variation, while relative volumes reveal how investment in specific neuropils changes independently of overall brain size. We found a significant positive relationship between total brain volume and head capsule volume (*R*^2^=0.12, *P*=0.036), indicating that individuals with larger heads tend to have larger brains. Head capsule volume did not vary significantly with age (*P*=0.745), suggesting that head size remains stable across the studied age range.

**Fig. 6.**
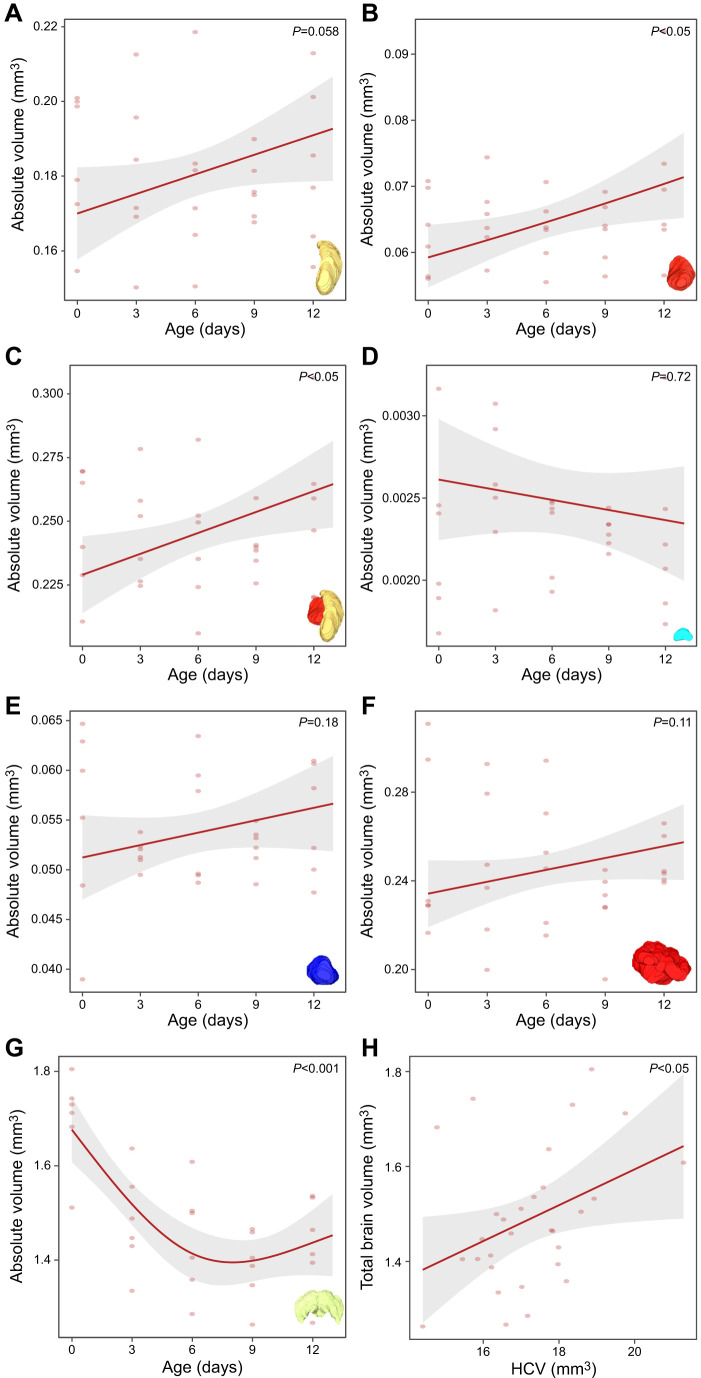
**Effect of age on the absolute volume of major neuropils in *V. vulgaris* (*n*=30).** Shown are model predictions from linear models (lm). (A) Medulla: marginally non-significant increase (*P*=0.058). (B) Lobula: significant increase (*P*<0.05). (C) Optic lobes: significant increase (*P*<0.05). (D) Central complex: no significant result (*P*=0.72). (E) Antennal lobes: no significant increase (*P*=0.18). (F) Mushroom bodies: no significant increase (*P*=0.11). (G,H) Total brain absolute volume: significant decrease with age (*P*<0.001) and significant increase with head capsule volume (HCV) (*P*<0.05). The 95% confidence intervals are given and each data point corresponds to an individual wasp.

Effects of age, head capsule volume (HCV) and total brain volume (TBV) were analysed on the absolute volumes of the major neuropils. For the medulla, TBV had a significant positive effect on volume (*F*_1,26_=11.44, *P*=0.002), while the effect of age was marginally non-significant (*F*_1,26_=3.93, *P*=0.058) and HCV had no significant effect (*F*_1,26_=0.0010, *P*=0.975) (Fig. 6A). For the lobula (log-transformed model), both age (*F*_1,26_=6.37, *P*=0.018) and TBV (*F*_1,26_=5.59, *P*=0.026) had significant positive effects, whereas HCV was not significant (*P*=0.62) (Fig. 6B). The OLs (medulla and lobula combined) showed a significant increase in volume with both age (*F*_1,26_=6.48, *P*=0.017) and TBV (*F*_1,26_=12.95, *P*=0.001), while HCV was again not a significant predictor (*P*=0.876) (Fig. 6C). In contrast, the volume of the central complex (spline model TBV) was not significantly affected by age (*P*=0.72), HCV (*P*=0.42) or TBV (*P*=0.94) (Fig. 6D). Similarly, antennal lobe volume showed no significant effect of age (*P*=0.18), HCV (*P*=0.49) or TBV (*P*=0.065) (Fig. 6E). For the MBs, both HCV (*F*_1,26_=9.36, *P*=0.005) and TBV (*F*_1,26_=11.03, *P*=0.003) had highly significant positive effects, while age was not significant (*P*=0.108) (Fig. 6F). Finally, TBV (spline model for age) itself decreased significantly with age (*F*_1,26_=19.16, *P*<0.001) and increased with HCV (*F*_1,26_=6.48, *P*=0.017) (Fig. 6G). Although age did not show a significant effect for most of these individual neuropils, there seemed to be a general increasing trend except for the central complex.

**Fig. 7.**
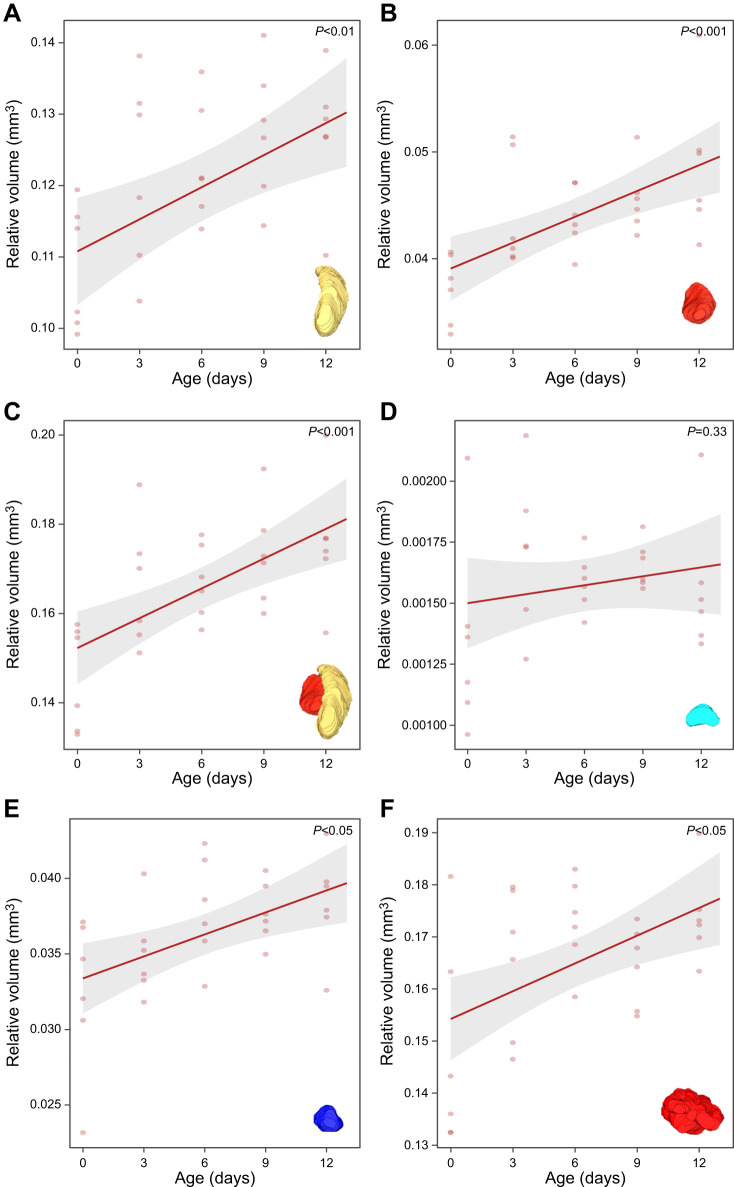
**Effect of age on the relative volume of major neuropils in *V. vulgaris* (*n*=30).** Shown are model predictions from linear models (lm). (A) Medulla: significant increase (*P*=0.002). (B) Lobula: significant increase (*P*<0.001). (C) Optic lobes: significant increase (*P*<0.001). (D) Central complex: no significant result (*P*=0.33). (E) Antennal lobes: significant increase (*P*=0.004). (F) Mushroom bodies: significant increase (*P*=0.002). The 95% confidence intervals are given and each data point corresponds to an individual wasp.

To examine whether this increasing trend is relevant, we investigated the effects of age and HCV on the relative volumes of these neuropils. We found a significant positive effect of age for the relative volume of all neuropils except the central complex. For the medulla (spline model for HCV), age led to a significant increase in relative volume (*F*_1,26_=10.21, *P*=0.004) (Fig. 7A), whilst HCV had no significant effect (*F*_1,26_=0.24, *P*=0.62). Age had a significant positive effect on the relative volume of the lobula (*F*_1,27_=16.37, *P*<0.001), whereas HCV was not significant (*P*=0.39) (Fig. 7B). The OLs showed a significant increase in relative volume with age (*F*_1,27_=16.88, *P*<0.001) and HCV was again not significant (*P*=0.47) (Fig. 7C). Similar to the absolute volume, the relative volume of the central complex was not significantly affected by age (*P*=0.33) or HCV (*P*=0.79) (Fig. 7D). Age also had a significant positive effect on the relative antennal lobe volume (*F*_1,27_=9.90, *P*=0.004) and HCV was not significant (*P*=0.78) (Fig. 7E). Lastly, the relative volume of the MBs was also positively significantly affected by age (*F*_1,27_=11.27, *P*=0.002) and HCV (*F*_1,26_=6.71, *P*=0.015) (Fig. 7F). Overall, all examined neuropils increased in relative volume with age, with the central complex as the sole exception; only the MBs showed an HCV effect.

## DISCUSSION

In this study, we investigated whether age influences learning and memory performance in workers of the eusocial wasp *V. vulgaris* and whether behavioural changes correspond with structural brain changes. While younger workers (0–3 days old) learned visual colour–reward associations more effectively than older foragers (9–12 days old), STM remained broadly preserved across ages, except for the oldest individuals. Neuroanatomical analyses revealed age-related shifts in relative neuropil volumes, suggesting brain plasticity associated with adult behavioural maturation.

### Age-related colour preference

We first asked whether wasps displayed an inherited bias towards particular colours, as such bias could influence associative learning outcomes. Initial colour bias varied with age. Newly emerged wasps (0–3 days old) strongly preferred blue, while older wasps (12 days old) favoured yellow. This transition probably reflects dynamic changes in perceptual or motivational state. A default bias toward short-wavelength colours, as documented in naive *Bombus* and *Apis* species (Briscoe and Chittka, 2001; Raine and Chittka, 2007), may explain the early blue preference. Older free-flying workers of honeybees and the common wasp also showed an initial preference for blue (Howard and Dyer, 2024). Alternatively, the relatively darker blue arm of the Y-maze may mimic nest-like conditions, which are preferred by young in-nest workers. The later emergence of a yellow bias could correspond to increased responsiveness to ecologically relevant cues associated with foraging (Papiorek et al., 2016), or simply to perceived brightness mimicking outdoor light conditions. This behavioural shift parallels structural changes in the brain, such as the relative volume of the OLs, the neuropil for visual processing, which significantly increased with age. Experience-dependent modulation of colour preference has been observed in other insects (Chittka and Raine, 2006; Muñoz-Galicia et al., 2021), suggesting that early neural development and the onset of foraging jointly shape perceptual bias.

### Evidence for age-related decline in learning

Our results indicate a pronounced age-related decline in colour–reward associative learning. Although all age groups have demonstrated visual associative learning, improving in accuracy and reducing search time across trials, consistent with appetitive conditioning studies in wasps and bees (Dyer and Garcia, 2014; Dyer and Howard, 2023; Giurfa, 2004), younger wasps reached higher accuracy than older workers, contrary to our initial hypothesis. This indicates that newly emerged wasps possess functional neural circuits for learning shortly after emergence, and that cognitive decline in older workers is more likely to be due to senescence than lack of experience. This is also consistent given the average longevity of an adult *V. vulgaris* wasp is 2 weeks (Spradbery, 1971). Spatial memory may have interfered with colour learning, particularly when maze arm positions were switched. As shown in *V. germanica*, wasps often rely on the spatial position of a reward rather than colour alone (Moreyra et al., 2017; Moreyra and Lozada, 2021). We switched the coloured arms to promote visual learning instead of spatial learning, which made it more challenging for wasps to find the reward. This was particularly observed in wasps trained under treatments Y2 and B2, where the maze configuration remained unchanged for three consecutive trials. Rapid choices toward previously rewarded positions in our trials suggest that older wasps may prioritise familiar spatial cues over new associations.

In contrast to finding an increase in learning ability with age, as found previously in *Mischocyttarus cerberus* (da Silva et al., 2023), we did not find evidence for increased learning ability in older workers. However, in *Polistes* wasps, young individuals exhibit early recognition of nestmates, even in the absence of olfactory cues (Signorotti et al., 2014). This could suggest an alternative trend of high learning ability early on, followed by an age-related cognitive decline as observed across animal taxa (Iliadi and Boulianne, 2010; Tonoki and Davis, 2015). Alternatively, older foragers may face cognitive constraints due to increased memory load (Moreyra and Lozada, 2021; Scheiner and Amdam, 2009). However, given the limited foraging range in our setup, true memory overload appears less likely, and reduced plasticity may instead reflect intrinsic age-related changes in brain function.

Furthermore, there seemed to be a difference in strategy between young and old workers, indicating a speed–accuracy trade-off. Older wasps completed trials faster but with lower accuracy, suggesting a shift toward a fast but error-prone decision strategy (Chittka et al., 2009; Stroeymeyt et al., 2010). Individual and within-individual speed–accuracy trade-offs have been previously found in bumblebees (Chittka et al., 2003). Such behaviour could reflect foraging-related pressures in natural contexts, where rapid decisions may outweigh perfect accuracy. In many social insects, ageing and task specialisation are deeply intertwined, complicating interpretation. For example, Scheiner and Amdam (2009) demonstrated that cognitive performance in honeybees is more closely tied to foraging experience than to chronological age. However, without controlling for individual foraging experience, the influence of experience versus age remains unclear (Ben-Shahar and Robinson, 2001).

In our study, we examined the effects of age on learning performance but did not directly assess task specialisation, as in *V. vulgaris* there is an increase in foragers related to age in wasps reared in captivity (Ferreira et al., 2023). Similarly, our experimental conditions may have promoted accelerated behavioural maturation; given the small artificial colony size and limited brood, workers may have transitioned to foraging roles more rapidly than under natural conditions. While this design allowed us to include newly emerged individuals, which would normally not perform foraging tasks, and to subject the wasps to similar environmental conditions, it lacked ecological validity compared with testing free-flying foragers. Furthermore, we used water as a neutral control, but using an aversive stimulus such as quinine could have enhanced learning performance, as shown in *Vespula* wasps (Dyer and Howard, 2023).

### STM remains largely intact with ageing

By contrast, STM formation appeared largely resilient to ageing effects. Despite lower learning performance, STM retention across 2 h was largely consistent among age groups, with only 12-day-old wasps failing to perform above chance. This suggests that STM remains relatively robust, although a declining trend at 6–9 days old was observed. These results mirror findings in honeybees and ants, where some cognitive functions remain resilient to ageing, while others, particularly new associative learning, decline (Behrends and Scheiner, 2010; Giraldo et al., 2016). Longer decision times predicted poor memory performance, with older wasps being faster but less accurate. This suggests individual, experience-based foraging decisions rather than coordinated recruitment (Lozada and D'Adamo, 2006, 2014; Moreyra et al., 2017), highlighting the importance of memory in their natural behaviour, regardless of age.

### Selective plasticity in the ageing brain

At the neural level, ageing was associated with increased MB and OL volumes, but these changes did not directly mirror cognitive performance. Despite the observed decline in the absolute total brain volume with age, our neuroanatomical analyses revealed the opposite trend at the structural level: relative volumes of most major neuropils (OL, AL, MB) increased, with the CX remaining stable. The same pattern was less noticeable in absolute volume, which showed only a non-significant positive trend with age, which suggests that age-related differences in relative investment are not driven by changes in total brain size. This pattern points to selective investment in sensory and integrative regions, probably reflecting the energetic costs of maintaining and expanding neural tissue involved in sensory processing or learning, which may in turn lead to compensatory reductions in less behaviourally relevant brain regions (Poissonnier et al., 2023). Similar region-specific plasticity has been reported in *Apis* and *Bombus*, where MB and OL growth is both age and experience dependent (Fahrbach et al., 1998; Jones et al., 2013; Withers et al., 1995). Work on solitary alkali bees further shows that experience rather than age enlarges MB subregions, reinforcing the primacy of adult experience (Hagadorn et al., 2021).

As *V. vulgaris* workers start off in the nest and only transition to foraging tasks after a few days (Ferreira et al., 2023), we expected regions such as the OL and MB to increase in volume with age. Interestingly, the high early learning ability in young workers implies that the MB and OL are already functionally competent before full structural maturity, consistent with findings in honeybees and *Polistes* wasps (Maleszka et al., 2009; Signorotti et al., 2014). Still, there was also a significant increase of the relative volume of the MB, similar to previous studies in honeybees, where the first week after emergence is marked by substantial structural plasticity in the MB (Fahrbach et al., 1998; Withers et al., 1995). In this species, MB growth occurs both through intrinsic developmental processes and through experience-dependent mechanisms linked to foraging (Fahrbach et al., 1998; Withers et al., 1995). However, MB expansion alone does not necessarily predict improved learning performance (Maleszka et al., 2009), which may explain why older wasps, despite having relatively larger neuropils, exhibited reduced learning capacity. Prior studies have found that increases in MB volume are more experience driven and not strictly age related, as larger MB expansion is observed in foragers compared with age-matched nurse bees (Maleszka et al., 2009). Comparable patterns of early MB maturation were observed in *Bombus* bumblebees and *Polistes* wasps (Jernigan et al., 2021; Jones et al., 2013), and may represent a common strategy among insects with shorter lifespans and accelerated task transitions, as is the case for the common wasp. Together with evidence from solitary alkali bees, these patterns suggest that experience-dependent MB plasticity is widespread and probably ancestral, while in complex eusocial species, the age-related division of labour makes neural maturation more predictably age correlated, driven by experience rather than chronological age per se (Fahrbach et al., 1998; Hagadorn et al., 2021).

Similarly to the MB, OL and AL showed a significant age-related change in relative volume, indicating that olfactory and visual processing centres are already developed early on in life, but seem to increase in importance with age. Again, this is similar to findings in honeybees where antennal lobe volume increased in an activity-dependent manner and was associated with improved associative learning performance (Sigg et al., 1997; Winnington et al., 1996). Analogous experience- and age-related expansion of visual neuropils has been documented in *Bombus* and *Apis* species, where increasing foraging activity drives sensory system plasticity (Fahrbach et al., 1998; Jones et al., 2013). Furthermore, Jernigan et al. (2021) also found that mature individuals had relatively larger visual regions of newly emerged workers in *P. fuscatus* (Jernigan et al., 2021). In contrast to all other brain regions, the CX did not vary with age, indicating that central integration centres remain stable across early adult development in *V. vulgaris*.

In conclusion, our study shows that in the common wasp, *V. vulgaris*, visual learning ability declines with age while STM remains comparatively stable. Although these findings did not support previous studies in *M. cerberus* showing increasing learning ability with age (da Silva et al., 2023), they do suggest that it is not chronological age but rather experience that controls learning and memory capacities (Scheiner and Amdam, 2009). Brain plasticity appears to be region specific, supporting early functional readiness followed by selective structural growth in visual and olfactory centres. Because of our laboratory setup, individuals experienced a simplified environment and restricted foraging opportunities compared with individuals under natural conditions. This reduced exposure to navigational, sensory and environmental challenges probably limited experience-driven neuroplasticity, particularly in brain regions such as the MBs or OLs. Studies in other social insects demonstrate that environmental complexity and experience strongly influence neural development: honeybee workers reared in social isolation and darkness show constrained MB growth (Fahrbach et al., 1998), and exposure to multisensory stimuli enhances brain development in *Bombus impatiens* (Jones et al., 2013). Additionally, foraging under natural conditions exposes workers to nutritional variability that can influence brain development, as pollen availability shapes brain amino acid profiles thought to support neurodevelopment and neural plasticity across early adulthood in honeybees (Gage et al., 2020). These factors may therefore moderate the magnitude of age-related changes in brain volume observed in our study. At the same time, the controlled conditions allowed us to monitor all age classes and disentangle age-related effects from variation arising through task specialisation or differing foraging experiences.

To our knowledge, this is the first study to simultaneously examine age-related variation in both learning and STM and their neuroanatomy correlates in *V. vulgaris*. By integrating behavioural and neuroanatomical perspectives, our results highlight wasps as a valuable comparative model for understanding cognitive ageing in social insects. More specifically, future work could examine LTM and incorporate appetitive–aversive conditioning, which could result in more apparent differences between differently aged workers. Controlling for task specialisation and foraging experience in a natural setup will also be critical for disentangling the roles of chronological age versus behavioural experience in shaping cognition. This will allow a critical test of whether cognitive ageing reflects a conserved constrained or an adaptive trajectory tuned by ecological demands and social organisation.
